# Medication Adherence After Acute Coronary Syndrome in Women Compared With Men: A Systematic Review and Meta-Analysis

**DOI:** 10.3389/fgwh.2021.637398

**Published:** 2021-02-22

**Authors:** Sophie H. Bots, Jose A. Inia, Sanne A. E. Peters

**Affiliations:** ^1^Laboratory for Experimental Cardiology, University Medical Center Utrecht, Utrecht University, Utrecht, Netherlands; ^2^Julius Center for Health Sciences and Primary Care, University Medical Center Utrecht, Utrecht University, Utrecht, Netherlands; ^3^Imperial College London, The George Institute for Global Health, London, United Kingdom

**Keywords:** acute coronary syndome, sex differences, medication adherence, cardiovasccular medicine, women

## Abstract

**Introduction:** Pharmacological treatment is an important component of secondary prevention in acute coronary syndrome (ACS) survivors. However, adherence to medication regimens is often suboptimal, reducing the effectiveness of treatment. It has been suggested that sex influences adherence to cardiovascular medication, but results differ across studies, and a systematic overview is lacking.

**Methods:** We performed a systematic search of PubMed and EMBASE on 16 October 2019. Studies that reported sex-specific adherence for one or more specific medication classes for ACS patients were included. Odds ratios, or equivalent, were extracted per medication class and combined using a random effects model.

**Results:** In total, we included 28 studies of which some had adherence data for more than one medication group. There were 7 studies for angiotensin-converting enzyme inhibitors (ACEIs) or angiotensin II receptor blockers (ARBs) (*n* = 100,909, 37% women), 8 studies for antiplatelet medication (*n* = 37,804, 27% women), 11 studies for beta-blockers (*n* = 191,339, 38% women), and 17 studies for lipid-lowering medication (*n* = 318,837, 35% women). Women were less adherent to lipid-lowering medication than men (OR = 0.87, 95% CI 0.82–0.92), but this sex difference was not observed for antiplatelet medication (OR = 0.95, 95% CI 0.83–1.09), ACEIs/ARBs (OR = 0.95, 95% CI 0.78–1.17), or beta-blockers (OR = 0.97, 95% CI 0.86–1.11).

**Conclusion:** Women with ACS have poorer adherence to lipid-lowering medication than men with the same condition. There are no differences in adherence to antiplatelet medication, ACEIs/ARBs, and beta-blockers between women and men with ACS.

## Introduction

Patients who survive an acute coronary syndrome (ACS) are at high risk of recurrent events (1–3). Secondary prevention through pharmacological therapy reduces the risk of recurrent events and mortality in this population (1, 2), but its effectiveness is attenuated by suboptimal patient adherence (4, 5). Poor adherence to medication regimens is an important obstacle in improving outcomes for ACS patients and has proven difficult to solve (6). Two large meta-analyses evaluating adherence to cardiovascular medication found that patient sex was an important factor in predicting adherence (7, 8). However, these meta-analyses did not investigate which sex was at higher risk of non-adherence.

There is some evidence on adherence to, for example, statins that suggests women have poorer adherence because they experience more adverse drug reactions (9), but a structured overview of the literature is still lacking. We performed a systematic review with meta-analyses on sex differences in adherence to cardiovascular medication in patients with ACS. We hypothesize that women, in general, have poorer adherence than men.

## Methods

### Terminology

It is important to recognize that sex and gender describe two different concepts. *Sex* refers to the biological differences between females and males, whereas *gender* refers to social differences between women and men. Both play an important role in health and disease, although through different mechanisms (10). This manuscript evaluates sex differences, meaning the linguistically correct terms to use would be “female” and “male.” However, all studies included in our review used the terms “women” and “men” to refer to patient sex, as is common in medical literature. We therefore also use the terms “women” and “men” to refer to patients of the female and male sex, respectively.

### Search Strategy and Selection Criteria

We searched both PubMed and EMBASE on 16 October 2019 using a pre-defined search term consisting of both text words and MeSH headings (Supplementary Files). The text words were limited to title and abstract only. The retrieved articles were screened by two independent reviewers who also resolved any conflicts that arose with help of a third reviewer, if necessary. The reference lists of relevant articles were screened for any additional articles. A modified version of the Newcastle–Ottawa Scale was used to assess the quality of included studies (Supplementary Files).

Only original research articles written in English that evaluated adherence at the individual patient-level were eligible for inclusion. Articles were included if they reported sex-specific data on medication adherence in patients with ACS, defined as either myocardial infarction or unstable angina (11). We excluded studies with too few participants to evaluate sex differences (*n* < 100), studies where ACS was included alongside other cardiovascular diseases and results could not be separated based on disease subgroup, and studies that included only men or only women. We also excluded studies that evaluated adherence to a combination of medications instead of per specific medication group. Finally, we excluded all studies for which the full text could not be retrieved.

We extracted population size, the percentage of women, mean age, total duration of follow-up, and measure of adherence used from all included studies. In addition, we extracted the number of adherent and non-adherent women and men or, if unavailable, unadjusted relative risk estimates (or equivalent). We also extracted adjusted relative risk estimates when available.

### Statistical Analysis

The meta-analysis was conducted conforming with the Meta-Analyses and Systematic Reviews of Observational studies (MOOSE) guidelines (12). We chose “good adherence,” as defined by each study, as our outcome and men as the reference category to facilitate interpretation of the results. We pooled the sex-specific odds ratios (ORs) using random effects meta-analysis because the included studies applied varying definitions of adherence and thus the estimated effect of sex on adherence can vary across these studies. In these situations, it is recommended to apply random effects meta-analysis instead of fixed effects meta-analyses (13).

We calculated the average sex-specific adherence across studies weighted by study size. We calculated unadjusted ORs for studies that presented number of adherent and non-adherent women and men. We converted the risk estimates from studies that used either a different outcome (poor adherence) or reference category (women) to fit our analysis. When studies stratified their analysis by subgroups, we pooled reported risk estimates using fixed effects meta-analysis and included the pooled risk estimate in our overall meta-analysis. We performed an additional analysis using only adjusted ORs to see whether adjustment would affect our crude estimates. We created funnel plots to check for publication bias.

All analyses were performed in R (R Core Team, Vienna, Austria). A *p* < 0.05 was considered statistically significant.

## Results

### Study Characteristics

**Figure 1:**
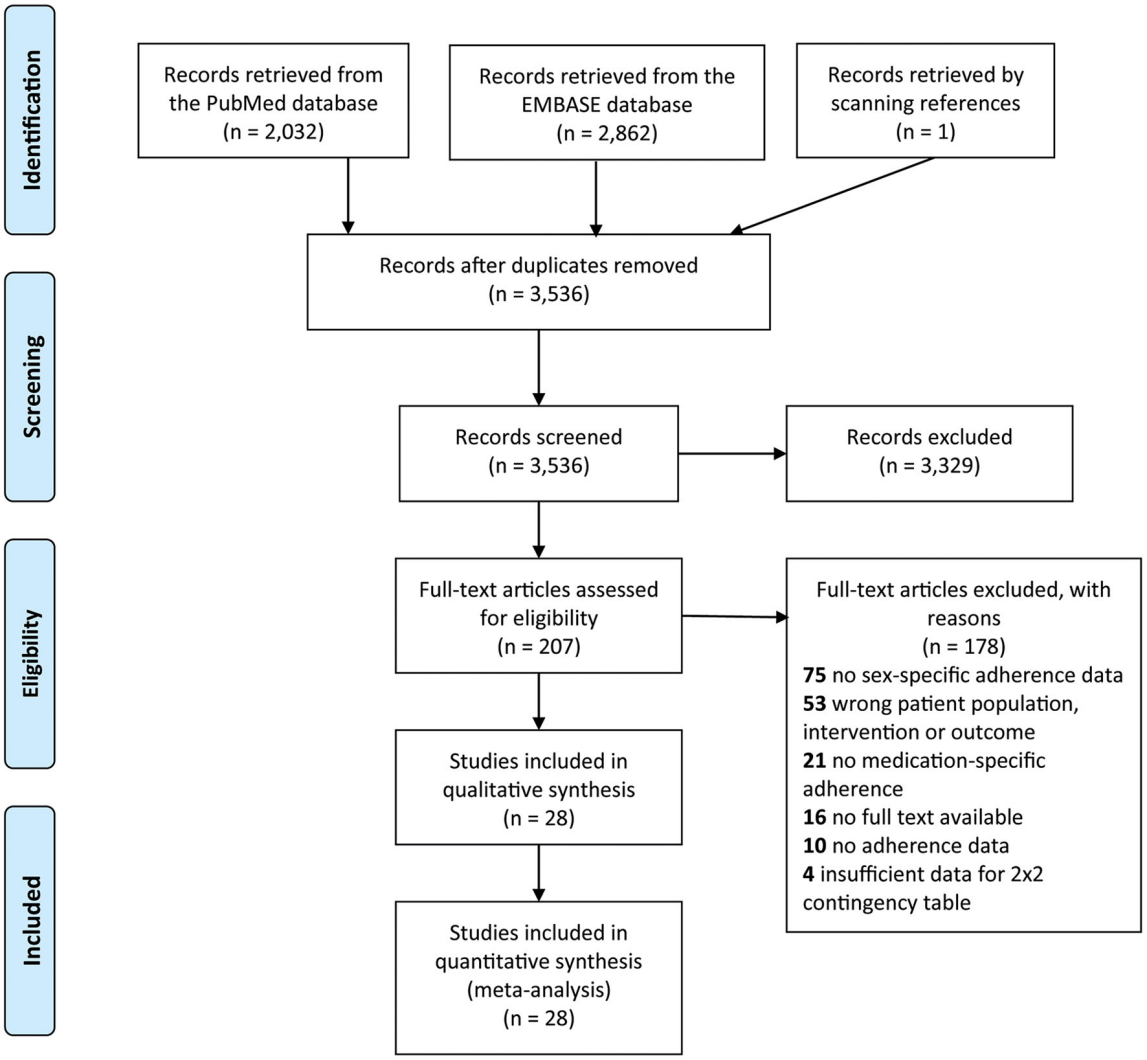
PRISMA 2009 Flow Diagram.

In total, we included 28 studies of which some had adherence data for more than one medication group (Figure 1). The medication groups included were angiotensin-converting enzyme inhibitors and angiotensin II receptor blockers (ACEIs/ARBs), antiplatelet therapy, beta-blockers, and lipid-lowering medication. Nine studies were of high quality (4 stars), 17 studies were of moderate quality (2 or 3 stars), and two studies were of poor quality (0 or 1 star). A complete overview of the included studies can be found in Table 1.

**Table 1:** Overview of studies included in systematic review and meta-analysis.

**First author (year of publication)**	**Study population**	**Country**	**Measure of adherence; good adherence**	**Medication groups evaluated**	**Number of participants (% women)**	**Quality**
Akincigil et al. (14)	Patients hospitalized with AMI	USA	Drug possession rate; ≤ 60 days elapsed between refills	Angiotensin system Beta-blocker	526 (36.7) 499 (33.3)	High
Allen LaPointe et al. (15)	Patients with high-risk NSTE-ACS	USA	Self-report; never missed a dose	Angiotensin system Beta-blocker Lipid-lowering	702 (31.9) 882 (30.5) 873 (31.0)	Moderate
Alsabbagh et al. (16)	Patients hospitalized with ACS	Canada	PDC; ≥80%	Lipid-lowering	9,051 (30.8)	High
Brogaard et al. (17)	Patients discharged with AMI diagnosis	Denmark	MPR; ≥80%	Lipid-lowering	1,024 (32.4)	High
Butler et al. (18)	Medicaid beneficiaries discharged with AMI diagnosis	USA	Maintain prescription	Beta-blocker	308 (54.9)	High
Colantonio et al. (19)	Medicare beneficiaries hospitalized for MI	USA	PDC; ≥80%	Lipid-lowering	29,125 (44.8)	Moderate
Degli-Espoti et al. (20)	Patients discharged with AMI diagnosis	Italy	Maintain prescription	Antiplatelet	5,919 (32.1)	High
Eagle et al. (21)	Patients with ACS	GRACE registry (14 countries)	Maintain prescription	Angiotensin system Antiplatelet Beta-blocker Lipid-lowering	2,364 (30.8) 12,393 (31.7) 7,686 (30.7) 6,277 (30.0)	Moderate
Fang et al. (22)	Medicare beneficiaries aged ≥65 years, alive 30 days after index AMI hospitalization	USA	PDC; ≥80%	Angiotensin system Beta-blocker Lipid-lowering	47,124 (59.0) 64,939 (57.5) 52,185 (55.9)	Moderate
Green et al. (23)	Patients admitted to hospital with first-time MI	Denmark	PPC	Antiplatelet	4,772 (33.0)	High
Hickson et al. (24)	Medicare beneficiaries aged >65 years with statin use prior to index AMI hospitalization	USA	PDC; ≥80%	Lipid-lowering	113,296 (54.3)	Moderate
Holme et al. (25)	Patients with history of confirmed AMI	IDEAL (6 North-European countries)	Total medication exposure/total study follow-up; ≥80%	Lipid-lowering	8,888 (19.1)	Moderate
Kramer et al. (26)	Patients hospitalized with AMI who survived for at least 1 year	USA	PDC; ≥75%	Beta-blocker	17,035 (29.5)	Moderate
Kubica et al. (27)	Patients treated with PCI for AMI	Poland	Quantity purchased/quantity prescribed; ≥80%	Antiplatelet	184 (25.0)	Moderate
Lauffenburger et al. (28)	Medicare beneficiaries aged ≥65 years, alive 30 days after index AMI hospitalization	USA	PDC; ≥75%	Angiotensin system Beta-blocker Lipid-lowering	46,286 (40.6) 63,856 (42.0) 51,321 (43.7)	Moderate
Luu et al. (29)	AMI patients who received coronary artery intervention	Vietnam	Self-report; never missed a dose	Antiplatelet	175 (27.0)	Poor
McGinnis et al. (30)	Kaiser Permanente Colorado beneficiaries with an incident coronary event	USA	PDC; ≥80%	Lipid-lowering	2,201 (29.4)	Moderate
Monaldi et al. (31)	Patients discharged with main diagnosis of MI	Italy	MPR; ≥80%	Lipid-lowering	2,629 (27.3)	Moderate
Nordstrom et al. (32)	Patients discharged from hospital after ACS-PCI	USA	MPR; ≥80%	Antiplatelet	1,340 (20.5)	Moderate
Ohlsson et al. (33)	Patients with discharge diagnosis of AMI	Sweden	Fill prescription within 3 months after discharge	Angiotensin system Lipid-lowering	1,346 (31.9) 1,346 (31.9)	Moderate
Phan et al. (34)	Kaiser Permanente South Colorado beneficiaries aged ≥80 years hospitalized for AMI	USA	PDC; ≥80%	Lipid-lowering	5,629 (50.0)	Moderate
Rasmussen et al. (35)	Patients aged >65 years who survived at least 1 year and 3 months after index AMI hospitalization	Canada	PDC; ≥80%	Beta-blocker Lipid-lowering	24,319 (44.3) 17,823 (41.9)	High
Sanfelix et al. (36)	Patients discharged after MI hospitalization	USA	Days when drug available; ≥75%	Beta-blocker	8,672 (28.5)	High
Sun et al. (37)	Patients diagnosed with ACS at discharge	China	PDC; ≥50%	Angiotensin system Antiplatelet Beta-blocker Lipid-lowering	2,561 (25.1) 3,318 (25.1) 2,757 (26.4) 3,648 (25.4)	Moderate
Turner et al. (38)	Patients discharged on high-intensity statin after index NSTE-ACS hospital admission	UK	Self-report; never missed a dose	Lipid-lowering	1,005 (24.2)	Poor
Wei et al. (39)	Patients who survived for 1 year after their first MI hospitalization	UK	MPR; ≥80%	Beta-blocker	386 (34.5)	High
Xie et al. (40)	Patients hospitalized with ACS who survived for 6 months without recurrent MI or stroke	China	Usage and dose over time; continuous use without dose decline	Lipid-lowering	12,516 (29.9)	Moderate
Zhu et al. (41)	MarketScan beneficiaries aged 18–65 years hospitalized with primary diagnosis of ACS who underwent PCI	USA	MPR; ≥80%	Antiplatelet	9,703 (22.4)	Moderate

*ACS, acute coronary syndrome; AMI, acute myocardial infarction; MPR, medication possession ratio; NSTE, non-ST elevation; PCI, percutaneous coronary intervention; PDC, proportion of days covered; PPC, proportion of patients covered; UK, United Kingdom; USA, United States of America*.

### Sex-Specific Adherence

In the crude analyses, we included 7 studies with adherence information for ACEIs/ARBs (*n* = 100,909, 37% women), 8 for antiplatelet medication (*n* = 37,804, 27% women), 11 for beta-blockers (*n* = 191,339, 38% women), and 17 for lipid-lowering medication (*n* = 318,837, 35% women). Across all included studies, 62.7% of women and 63.9% of men had good ACEIs/ARBs adherence. These percentages were 64.5 and 66.0% for antiplatelet medication, 60.8 and 61.0% for beta-blockers, and 73.6 and 75.3% for lipid-lowering medication, respectively.

**Figure 2:**
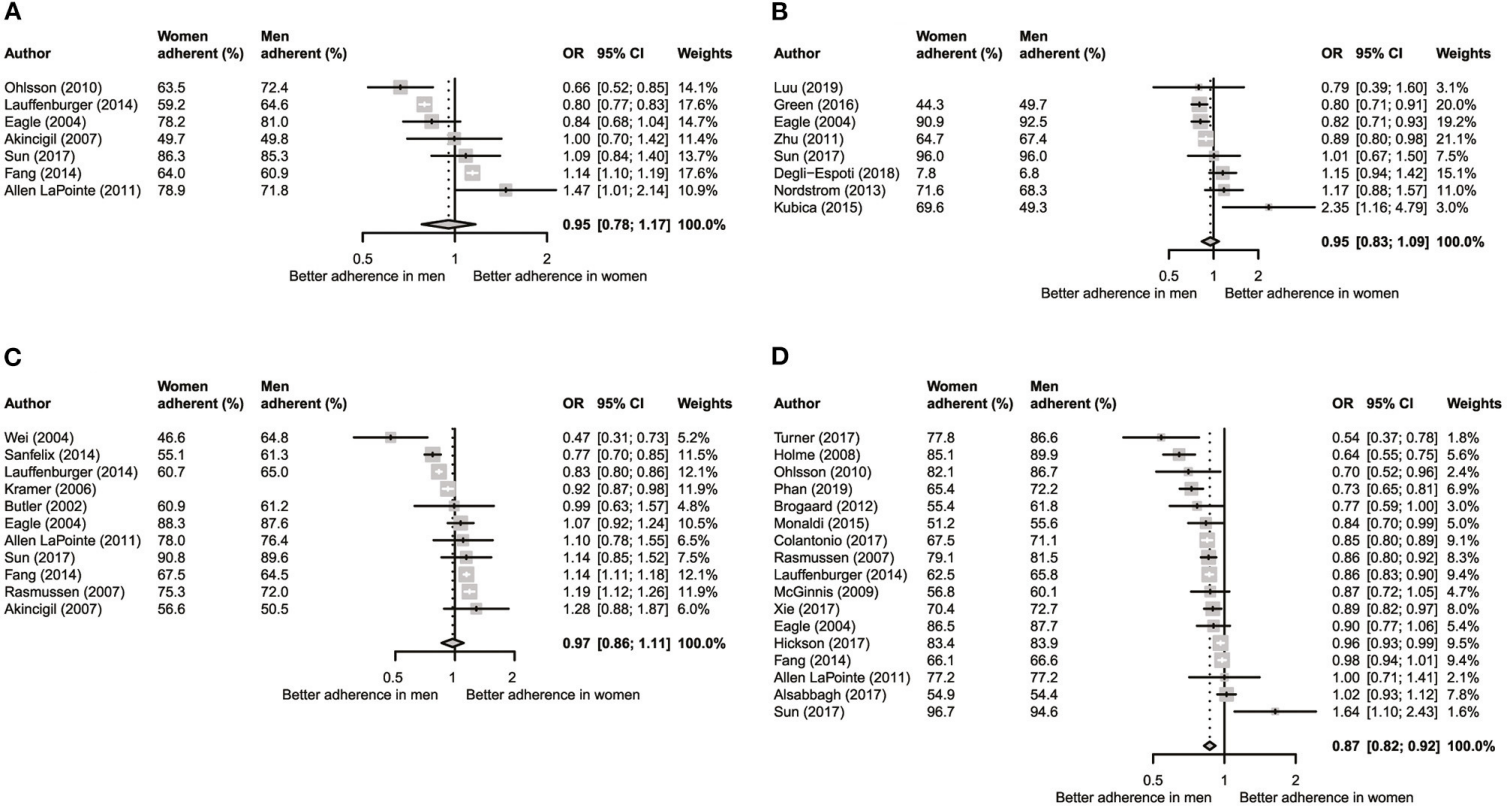
Meta-analysis of unadjusted odds ratios per medication category. **(A)** Angiotensin-converting enzyme inhibitors and angiotensin II receptor blockers. **(B)** Anti-platelet medication. **(C)** Beta-blockers. **(D)** Lipid-lowering medication.

There was no significant sex difference in adherence for ACEIs/ARBs (OR = 0.95, 95% CI 0.78–1.17; Figure 2A), antiplatelet medication (OR = 0.95, 95% CI 0.83–1.09; Figure 2B), and beta-blockers (OR = 0.97, 95% CI 0.86–1.11; Figure 2C). However, women had significantly poorer adherence to lipid-lowering medications than men (OR = 0.87, 95% CI 0.82–0.92; Figure 2D).

**Figure 3:**
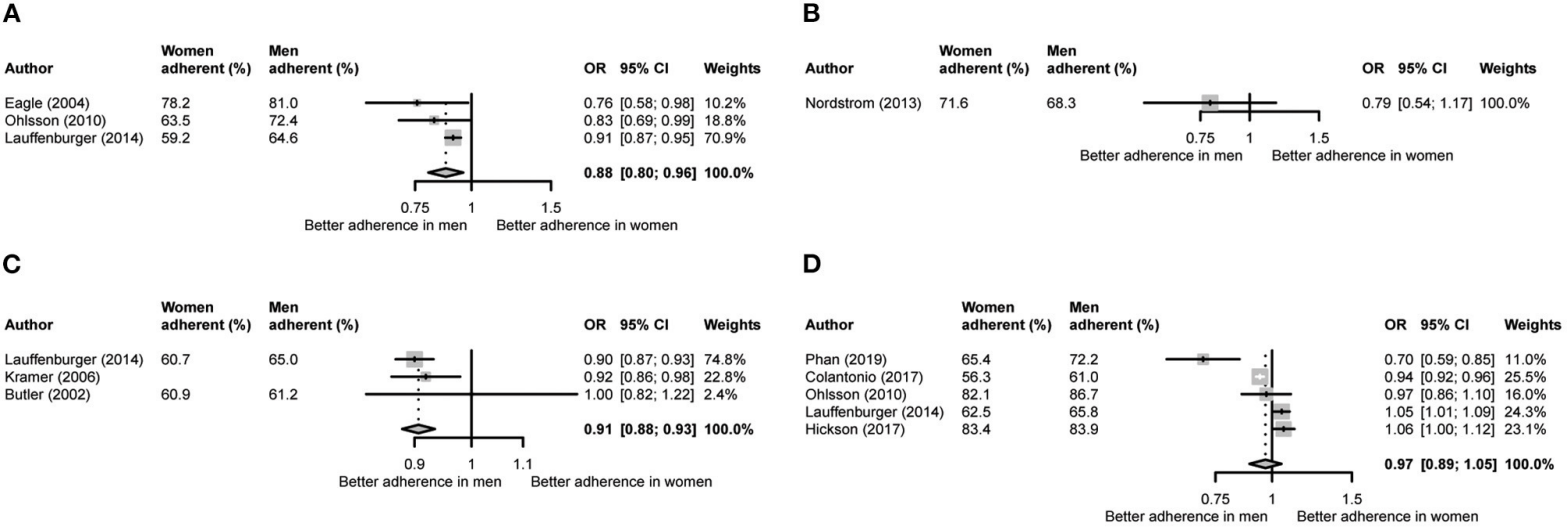
Meta-analysis of adjusted odds ratios per medication category. **(A)** Angiotensin-converting enzyme inhibitors and angiotensin II receptor blockers. **(B)** Anti-platelet medication. **(C)** Beta-blockers. **(D)** Lipid-lowering medication.

In the adjusted analyses, we included three studies for ACEIs/ARBs and beta-blockers and six studies for lipid-lowering medication. There was only one study with adjusted risk estimates for antiplatelet medication. These analyses showed a significantly poorer adherence in women for ACEIs/ARBs (OR = 0.88, 95% CI 0.80–0.96) and beta-blockers (OR = 0.91, 95% CI 0.88–0.93) but not for lipid-lowering medication (OR = 0.97, 95% CI 0.89–1.05) (Figure 3).

**Figure 4:**
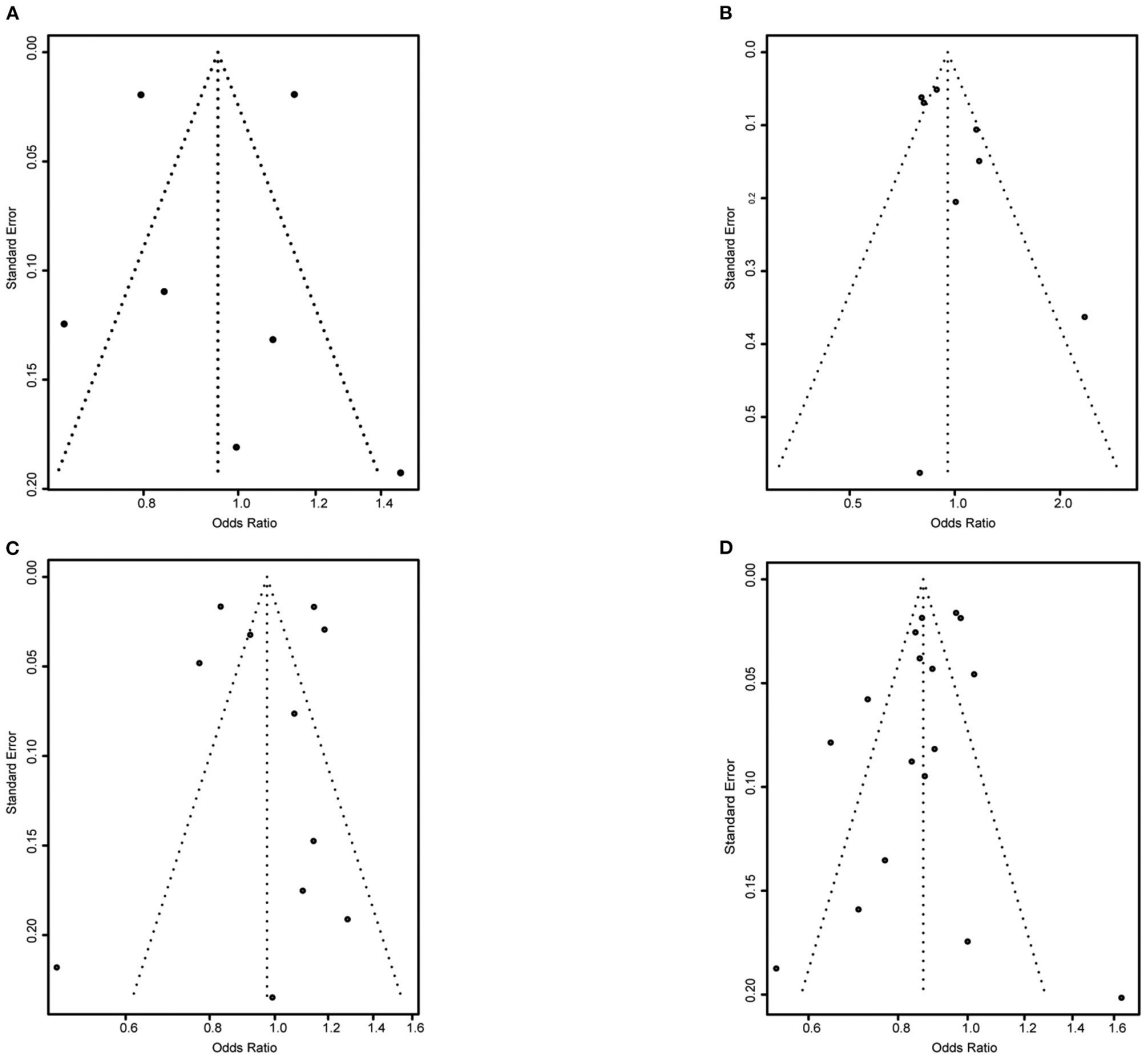
Funnel plot for each medication category. **(A)** Angiotensin-converting enzyme inhibitors and angiotensin II receptor blockers. **(B)** Anti-platelet medication. **(C)** Beta-blockers. **(D)** Lipid-lowering medication.

The funnel plots for ACEIs/ARBs and lipid-lowering medication were relatively balanced, suggesting little publication bias. For antiplatelet medication and beta-blockers, however, smaller studies showing poorer adherence in women seemed to be lacking compared with the number of such studies showing poorer adherence in men (Figure 4).

## Discussion

Adherence to ACEIs/ARBs, antiplatelet medication, beta-blockers, and lipid-lowering medication lies between 60 and 70% in both women and men surviving an ACS. Women had poorer adherence than men for lipid-lowering medication but not for the other medication groups, where adherence was similar between the sexes.

A previous systematic review on adherence to cardiovascular medication in coronary heart disease patients also found adherence to be 60–70% (42), suggesting that adherence is reasonable in secondary prevention of ACS. However, they did not find any sex differences (42), whereas our results suggest that those may be present at least for lipid-lowering drugs. This finding is supported by previous work showing that women have poorer adherence to statins in both primary and secondary prevention (43). This may be due to biological or social reasons, or a combination of both. There are known biological differences in drug metabolism between women and men (44), which may increase the risk of statin-related adverse drug reactions in women (45). This may also be true for the other medication groups included in our review, but the lack of sex-specific data on medication efficacy, safety, and metabolism prevents researchers from drawing sound conclusions on this topic (46–48). Gender differences may also play a role in adherence, with women for example more often refusing or discontinuing statins because they do not believe the medication is safe (49). Given that women derive equal benefit from statin therapy as men (50), it is important to improve statin adherence in women through both collecting more high-quality sex-specific data on this topic and adapting treatment to individual patients by for example using lower dosages to reduce the risk of side effects (45).

The main strength of this review is that it combines data from 28 studies. However, it is limited by the quality of the available data. The majority of studies included in this review were of moderate quality, and the data were heterogeneous on several important points. Both the chosen measure of adherence and the definition of “good adherence” varied greatly across studies. Approximately half of the included studies used a standardized measure of adherence, such as the medication possession ratio (18% of studies) or proportion of days covered (36%), but others used either self-report (11%) or another, sometimes self-devised, measure (35%). This makes meta-analyzing such data and interpreting the results difficult. To alleviate this issue, it is important that future studies use both standardized measures of adherence and standardized cut-off values to denote good and poor adherence. We also saw that smaller studies showing poorer adherence in women were less likely to be published, and that studies showing poorer adherence in women more often provided adjusted risk estimates. This differential approach may introduce bias in meta-analyses such as ours and complicate the interpretation of our findings.

In conclusion, we show that adherence to cardiovascular medication is reasonable in women and men surviving an ACS. Women had poorer adherence to lipid-lowering medication than men, but this difference was not observed for the other cardiovascular medication groups. However, a standardized approach to the measurement and evaluation of adherence is needed to improve the quality of research performed in this field.

## Data Availability Statement

The original contributions presented in the study are included in the article/Supplementary Material, further inquiries can be directed to the corresponding author/s.

## Author Contributions

SB and JI performed the systematic search. SB analyzed the data and wrote the manuscript. SP conceived the project, supported data analyses, and critically reviewed the manuscript. All authors contributed to the article and approved the submitted version.

## Conflict of Interest

The authors declare that the research was conducted in the absence of any commercial or financial relationships that could be construed as a potential conflict of interest.
