# Supplementary material for: Medication Adherence After Acute Coronary Syndrome in Women Compared With Men: A Systematic Review and Meta-Analysis
Source: Front Glob Womens Health. 2021 Feb 22;2:637398. doi: 10.3389/fgwh.2021.637398 (PMC8594018; doi:10.3389/fgwh.2021.637398)
Supplement: Supplementary file 1 [file Table_1.DOCX]

## 1. Search strategy

For this review, studies were identified using PubMed and EMBASE.

1. Strategy to identify adherence to medication

medication adherence [Title/Abstract] OR medication nonadherence [Title/Abstract] OR medication non-adherence [Title/Abstract] OR medication compliance [Title/Abstract] OR medication noncompliance [Title/Abstract] OR medication non-compliance [Title/Abstract] OR medication persistence [Title/Abstract] OR medication nonpersistence [Title/Abstract] OR medication non-persistence [Title/Abstract] OR medication continuation [Title/Abstract] OR medication discontinuation [Title/Abstract] OR patient compliance* [Title/Abstract] OR patient noncompliance [Title/Abstract] OR patient non-compliance [Title/Abstract] OR patient adherence [Title/Abstract] OR patient nonadherence [Title/Abstract] OR patient non-adherence [Title/Abstract] OR treatment compliance [Title/Abstract] OR treatment adherence [Title/Abstract] OR therapeutic compliance [Title/Abstract] OR proportion of days covered [Title/Abstract] OR PDC [Title/Abstract] OR medication adherence [MeSH Terms] OR medication compliance [MeSH Terms] OR medication persistence [MeSH Terms] OR patient compliance [MeSH Terms]

1. Strategy to identify relevant diseases

acute coronary syndrom* [Title/Abstract] OR ACS [Title/Abstract] OR heart attack [Title/Abstract] OR myocardial infarct* [Title/Abstract] OR MI [Title/Abstract] OR AMI [Title/Abstract] OR STEMI [Title/Abstract] OR NSTEMI [Title/Abstract] OR ST elevat* [Title/Abstract] OR coronary thrombo* [Title/Abstract] OR coronary occlusion [Title/Abstract] OR unstable angina [Title/Abstract] OR unstable angina pectoris [Title/Abstract] OR myocardial ischemia* [Title/Abstract] OR myocardial ischaemia* [Title/Abstract] OR coronary artery disease* [Title/Abstract] OR major coronary event* [Title/Abstract] OR coronary heart disease* [Title/Abstract] OR coronary disease* [Title/Abstract] OR ischemic heart disease [Title/Abstract] OR ischaemic heart disease [Title/Abstract] OR acute coronary syndrome [MeSH Terms] OR myocardial infarct* [MeSH Terms]

1. Strategy to identify sex

sex[Title/Abstract] OR gender[Title/Abstract] OR male[Title/Abstract] OR men[Title/Abstract] OR man[Title/Abstract] OR female[Title/Abstract] OR women[Title/Abstract] OR woman[Title/Abstract] OR sex factors[MeSH Terms] OR male[MeSH Terms] OR female[MeSH Terms]

Use of an asterisk indicates an open ended search term. Parts i, ii, and iii were combined using ‘AND’ to search PubMed and each part was specifically translated for searching EMBASE.

## 2. Newcastle-Ottawa quality assessment scale

Note: a study can be awarded a maximum of one star for each numbered item. A maximum of one star can be given for Selection and a maximum of 3 stars can be given for Exposure.

**Selection**

1. Representativeness of the exposed cohort
   1. Truly representative of the average in the community *
   2. Somewhat representative of the average in the community *
   3. Selected group of users, e.g. nurses, volunteers
   4. No description of the derivation of the cohort

**Exposure**

1. Assessment of outcome
   1. Independent blind assessment *
   2. Record linkage *
   3. Self-report
   4. No description
2. Was follow-up long enough for outcomes to occur?
   1. Yes, minimally 6 months *
   2. No
3. Adequacy of follow-up of cohorts
   1. Complete follow-up – all subjects accounted for *
   2. Subjects lost to follow-up unlikely to introduce bias – small number lost >…% *
   3. Follow-up rate <…? and no description of those lost
   4. No statement

Studies would get a point for each *
